# Decrease in *ITGA7* Levels Is Associated with an Increase in α-Synuclein Levels in an MPTP-Induced Parkinson’s Disease Mouse Model and SH-SY5Y Cells

**DOI:** 10.3390/ijms222312616

**Published:** 2021-11-23

**Authors:** Sangeun Han, Min Hyung Seo, Sabina Lim, Sujung Yeo

**Affiliations:** 1 Department of Meridian and Acupoint, College of Korean Medicine, Kyung Hee University, Seoul 02453, Korea; sadgc0303@khu.ac.kr; 2 Department of Meridian and Acupoint, College of Korean Medicine, Sang Ji University, Wonju 26339, Korea; cstcl@naver.com; 3 Research Institute of Korean Medicine, Sang Ji University, Wonju 26339, Korea

**Keywords:** Parkinson’s disease, *ITGA7*, alpha-synuclein, MPTP, substantia nigra

## Abstract

We investigated the potential association between *integrin α7* (*ITGA7*) and alpha-synuclein (α-syn) in a methyl-4-phenyl-1,2,3,6-tetrahydropyridine (MPTP)-induced Parkinson’s disease (PD) mouse model. Tyrosine hydroxylase (TH), *ITGA7*, and α-syn expression in the substantia nigra (SN) of the brain were observed to examine the pathological characteristics of PD. To determine the relationship between *ITGA7* and PD, the expression of TH and α-syn was investigated after *ITGA7* siRNA knockdown in SH-SY5Y cells. The *ITGA7* microarray signal was decreased in the SN of the MPTP group, indicating reduced *ITGA7* expression compared to that in the control. The expression patterns of *ITGA7* in the control group and those of α-syn in the MPTP group were similar on immunohistochemical staining. Reduction in *ITGA7* expression by *ITGA7* siRNA administration induced a decrease in TH expression and an increase in α-syn expression in SH-SY5Y cells. The decreased expression of *ITGA7* significantly decreased the expression of bcl2 and increased the bax/bcl2 ratio in SH-SY5Y cells. These results suggest that reduced *ITGA7* expression may be related to increased α-syn expression and apoptosis of dopaminergic cells in an MPTP-induced PD mouse model. To the best of our knowledge, this is the first study to show an association between *ITGA7* and PD.

## 1. Introduction

As life expectancy and the prevalence of degenerative brain diseases increase, the number of patients with Parkinson’s disease (PD), the second most common degenerative brain disease after Alzheimer’s disease, is increasing rapidly [1]. The main symptoms of PD are motor function-related symptoms, including slow movement, tremor, rigidity, and postural instability [2]. PD is characterized by the loss of dopamine-secreting neurons in the substantia nigra (SN) located in the midbrain, but the cause of this decrease is still unknown [3]. The major histopathological feature of PD is the deposition of Lewy bodies, and the major protein component of these intracellular deposits is a fibrillar aggregate of α-synuclein (α-syn) [4]. Increased expression of α-syn was observed in both patients with PD and PD animal models and is reported to be closely related to PD pathology [5]. Self-aggregation of these proteins leads to the formation of an amphipathic helical structure, which may result in membrane association [6].

*Integrin α7* (*ITGA7*), also known as the integrin subunit alpha 7 and integrin alpha 7 chain 3, encodes an extracellular matrix (ECM)-binding protein [7]. Integrins are a major family of cell surface receptors that mediate ECM adhesion and are closely implicated in the regulation of various cellular functions, including embryonic development, tumor cell growth and metastasis, and programmed cell death [8]. This protein functions as a receptor for laminin-1, a basement membrane protein on the surface of skeletal myoblasts and muscle fibers [9].

Abnormal integrin expression is associated with several human diseases [10]. Defects in *ITGA7* are associated with congenital myopathy [11]. *ITGA7*-deficient mice display significant hyperplasia, hypertrophy of arteries and arterioles, and malformation of skeletal muscles [12,13]. In addition, *ITGA7* deficiency is common in muscular dystrophy and myopathy [14]. α-syn and integrin were first reported in 2005, in a case of MSA, another synucleinopathy [15], and there are also recent studies reporting integrins and α-syn [16,17].

Based on these previous studies, we hypothesized that *ITGA7* expression is involved in the pathological changes in PD. The main symptoms of PD are motor function-related symptoms; therefore, we speculated that the motor function-related symptoms of PD may be related to myopathy due to a decrease in *ITGA7* expression. Herein, we report that *ITGA7* expression is reduced in the SN in a 1-methyl-4-phenyl-1,2,3,6-tetrahydropyridine (MPTP)-induced Parkinsonism mouse model. We further showed that the reduction in *ITGA7* expression induces an increase in α-syn expression in SH-SY5Y cells treated with MPP+. To the best of our knowledge, this is the first study to show an association between *ITGA7* and PD.

## 2. Results

### 2.1. Reduction in Tyrosine Hydroxylase Expression in a Mouse Model of Chronic MPTP-Induced PD

**Figure 1:**
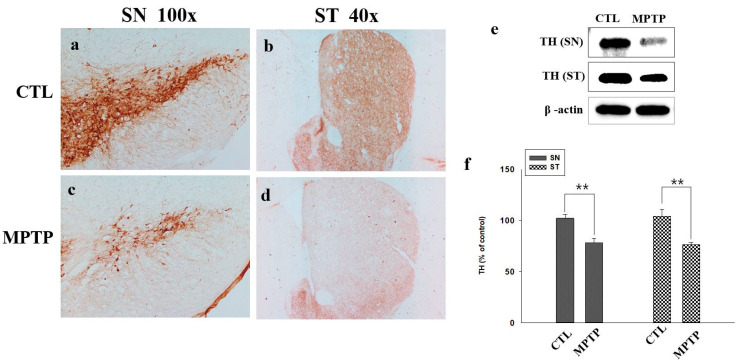
Immunohistochemistry analysis of tyrosine hydroxylase (TH) expression in the substantia nigra (SN; **a**,**c**) and the striatum (ST; **b**,**d**) in the control (CTL) and 1-methyl-4-phenyl-1,2,3,6-tetrahydropyridine-treated (MPTP) groups. TH expression decreased in the ST and SN regions of the brains of MPTP-treated mice. Western blot analyses (**e**) showed that TH expression was significantly decreased in MPTP-treated mice (**f**). ** denotes *p* < 0.005 compared to CTL. All values are expressed as mean ± standard error, and statistical analysis was performed using Student’s *t*-test.

MPTP and saline were injected intraperitoneally once daily in the control (CTL) and MPTP groups, respectively. After four weeks, to confirm that a chronic MPTP-induced PD mouse model was established, we analyzed the changes in tyrosine hydroxylase (TH) expression in the SN and striatum (ST). The expression of TH was significantly decreased in MPTP mice (Figure 1c,d) compared to that in the CTL mice in both the SN and ST. Similar to the results of the Western blot analysis, it was confirmed that the expression of TH was significantly reduced in the SN and ST (*p* < 0.005) treated with MPTP (Figure 1).

### 2.2. Microarray Analysis and Western Blot Analysis of *ITGA7* in the Substantia Nigra

**Figure 2:**
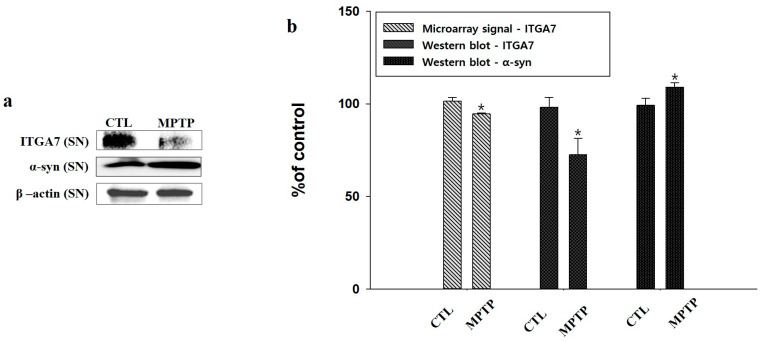
Integrin alpha 7 (*ITGA7*) expression was decreased and α-synuclein (α-syn) expression was increased in the MPTP group (MPTP) compared to that in the control group (CTL) in the SN. (**a**) Western blot analysis verified the decreased expression of *ITGA7* and the increased expression of α-syn in MPTP-treated mice. (**b**) Histogram shows a significant decrease in *ITGA7* expression, detected by Western blot analysis, and the decreased signal of microarray in the MPTP group compared to that in the CTL group. Histogram shows a significant increase in α-syn in the MPTP group compared to that in the CTL group. * denotes *p* < 0.05 compared to CTL. All values are expressed as mean ± standard error, and statistical analysis was performed using Student’s *t*-test.

Microarray signal analysis of the SN confirmed that the *ITGA7* signal in MPTP-treated mice was decreased compared to that in CTL (*p* < 0.05, *n* = 2, Figure 2b). Therefore, when *ITGA7* expression was evaluated by Western blot analysis, it was confirmed that the expression of *ITGA7* was significantly decreased in the MPTP group compared to that in the CTL group (*p* < 0.05, *n* = 3, Figure 2a,b). α-syn expression was evaluated using Western blot analysis, and it was confirmed that the expression of α-syn was significantly increased in the MPTP group compared to that in the CTL group (*p* < 0.05, *n* = 3, Figure 2a,b).

### 2.3. Reduction in *ITGA7* Expression and Increase in α-Syn Expression in the Substantia Nigra in a Mouse Model of Chronic MPTP-Induced PD

**Figure 3:**
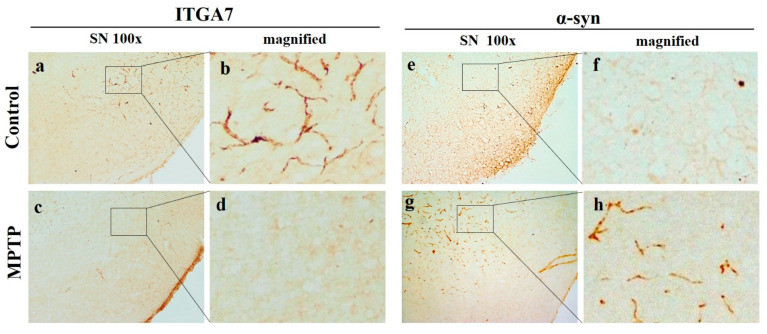
Representative images of (*ITGA7*) and α-synuclein (α-syn) expression in the SN in a chronic 1-methyl-4-phenyl-1,2,3,6-tetrahydropyridine (MPTP)-induced Parkinson’s disease mouse model. The expression of *ITGA7* (**a**–**d**) was decreased (**c**,**d**) and the expression of α-syn (**e**–**h**) was increased (**g**,**h**) in in the MPTP group. Images of (**b**,**d**,**f**,**h**) are magnified images of squares in (**a**,**c**,**e**,**g**).

Immunohistochemical analysis (IHC) showed that the expression of *ITGA7* in the SN was significantly decreased in the MPTP group (Figure 3c,d) when compared to that in the CTL group (Figure 3a,b). Conversely, the expression of α-syn was remarkably increased in the MPTP group (Figure 3e–h). Although the expression of *ITGA7* and α-syn was inversely proportional, the patterns were similar.

### 2.4. Immunofluorescence Analysis of *ITGA7* Co-Localized with a α-Syn in the Substantia Nigra

**Figure 4:**
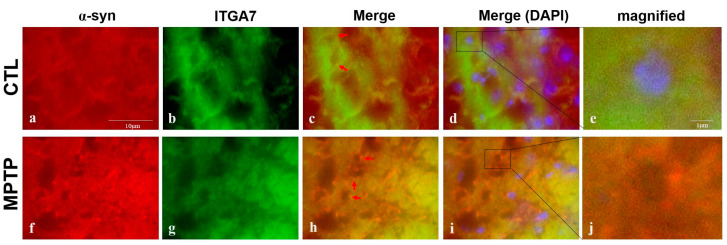
Immunofluorescence images of *ITGA7* co-localized with α-syn in the SN in an MPTP-induced Parkinson’s disease mouse model. SN regions were immunofluorescently labeled with anti-*ITGA7* (**b**,**g**) and anti-α-syn (**a**,**f**) antibodies by using Rhodamine Avidin (**a**,**f**, red) and were then double immunolabeled with *ITGA7* antibodies by using Fluorescein Avidin (**b**,**g**, green). The middle panels (**c**,**h**) show merged images of the individual α-syn (**a**,**f**) and *ITGA7* (**b**,**g**) panels. In the control group (**c**), *ITGA7* was more strongly expressed (green) and in the MPTP group (**h**), intense red fluorescence was observed. (**d**,**i**) show merged images with DAPI. Images of (**e**) and (**j**) show enlarged images of the square in (**d**) and (**i**). (scale bar, 10 µm).

Immunofluorescence analysis of *ITGA7* and α-syn revealed co-localization and showed that *ITGA7* expression was stronger in the CTL group (Figure 4a) than in the MPTP group (Figure 4f). Conversely, α-syn was more strongly expressed in the MPTP group than in the CTL group (Figure 4b,g). When *ITGA7* and α-syn were merged (Figure 4c,d,h,i), the expression of *ITGA7* was stronger in the CTL group (Figure 4d), whereas the expression of α-syn was stronger in the MPTP group (Figure 4i).

### 2.5. Western Blot Analysis in SH-SY5Y Cells Induced by *ITGA7* siRNA

**Figure 5:**
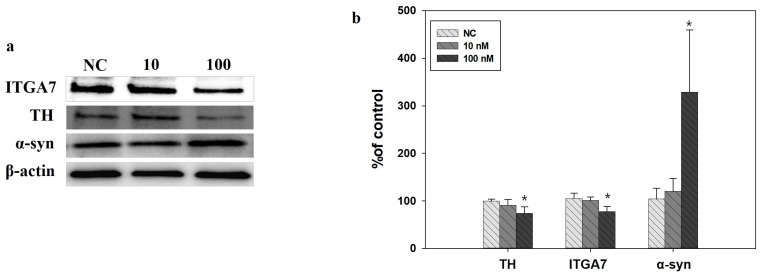
**(a)** Western immunoblot analysis showed that the administration of *ITGA7* siRNA decreased *ITGA7* and TH expression but increased α-synuclein (α-syn) expression in the SH-SY5Y cells. (**b**) NC, negative control siRNA treatment (100 nM for 24 h); 10, *ITGA7* siRNA treatment (10 nM for 24 h); 100, *ITGA7* siRNA treatment (100 nM for 24 h). * denotes *p* < 0.05 compared to NC. All values are expressed as mean ± standard error, and statistical analysis was performed using ANOVA.

**Figure 6:**
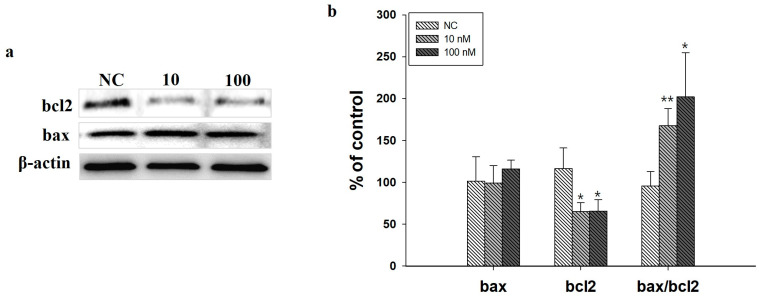
**(a)** Western immunoblot analysis revealed that the administration of *ITGA7* siRNA significantly decreased bcl2 expression and increased the bax/bcl2 ratio in SH-SY5Y cells. (**b**) NC, negative control siRNA treatment (100 nM for 24 h); 10, *ITGA7* siRNA treatment (10 nM for 24 h); 100, *ITGA7* siRNA treatment (100 nM for 24 h). * denotes *p* < 0.05 and ** *p* < 0.001 compared to NC. All values are expressed as mean ± standard error, and statistical analysis was performed using ANOVA.

*ITGA7* siRNA administration decreased *ITGA7* and TH expression in SH-SY5Y cells (*p* < 0.05, *n* = 3) but increased α-syn expression (*p* < 0.05, *n* = 3) (Figure 5a,b). In addition, *ITGA7* siRNA administration reduced the expression of bcl2 (*p* < 0.05), while bax expression did not increase significantly, resulting in a significantly increased bax/bcl2 ratio (Figure 6).

### 2.6. Immunofluorescence Analysis of *ITGA7* Revealed Co-Localization with α-Syn in SH-SY5Y Cells

**Figure 7:**
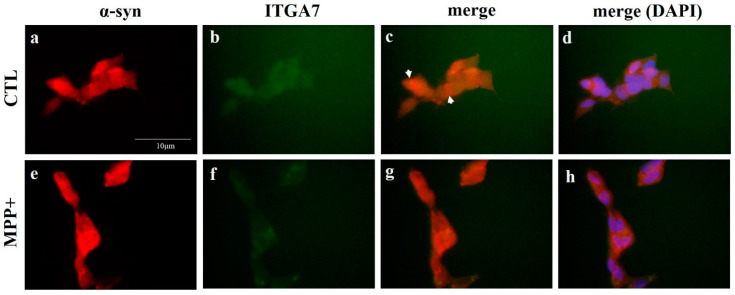
Immunofluorescence analysis of *ITGA7* co-localized with α-syn in SH-SY5Y cell. The cells were immunofluorescently labeled with anti-*ITGA7* (**b**,**f**) and anti-α-synuclein (**a**,**e**) antibodies using Rhodamine Avidin (**a**,**e**, red) and were then double immunolabeled with *ITGA7* antibodies using Fluorescein Avidin (**b**,**f**, green). The panels on the right (**c**,**g**) show merged images of the individual left (**a**,**e**) and middle (**b**,**f**) panels. (**d**,**h**) show merged images with DAPI (scale bar, 10 µm). White arrows indicate regions of interest.

Immunofluorescence analysis of *ITGA7* and α-syn revealed their co-localization, and the expression of *ITGA7* was stronger in the CTL than in MPP+-treated SH-SY5Y cells (Figure 7b,f). Conversely, the expression of α-syn was stronger in the MPP+-treated group than in the CTL group (Figure 7a,e). When α-syn and *ITGA7* were combined, *ITGA7* expression was strong in the CTL group and α-syn was strongly expressed in the MPP+-treated group (Figure 7c,g).

## 3. Discussion

It has previously been reported that an increase in α-syn expression is an important step in the pathology of PD [18,19], and our results suggest that one of the causes of this increase may be a decrease in *ITGA7* expression. The mechanism by which dopaminergic cell destruction occurs following increased α-syn expression has been reported to be related to its effect on membrane fusion machinery, which regulates neurotransmitter release [20,21]. α-syn is highly concentrated at the presynaptic nerve terminals in both soluble and membrane-bound states [22,23]. Membrane binding by α-syn is likely physiologically important because α-syn remodels membranes [24]. Moreover, membranes were found to be important for the neuropathological effects of α-syn. Importantly, several studies have shown the involvement of aberrant α-syn-membrane interactions in cytotoxicity [19]. The self-aggregation of α-syn promotes membrane disruption, which can result from the formation of an amphipathic helical structure of α-syn [6]. Modern views of PD encompass the cell-to-cell trans-synaptic spread of α-syn [25,26] and the existence of many α-syn polymorphs that might be responsible for different synucleinopathies [27,28]. The cell membrane containing integrins is thought to play an important role in the intercellular synaptic diffusion of α-syn. Although studies have shown the link between PD and integrins [15,29], studies in Chinese [30], Swedes [31]**,** and Taiwanese [32] reported that *ITGA8* was not associated with PD.

*ITGA7* encodes the ECM-binding protein. Integrins are a major family of cell surface receptors that mediate ECM adhesion and are strongly implicated in the regulation of various cellular functions, including programmed cell death [33]. Consistent with these previous studies, our results demonstrated that decreased *ITGA7* expression induced by *ITGA7* siRNA administration decreased TH expression in SH-SY5Y cells but increased α-syn expression. In addition, the decreased expression of *ITGA7* reduced the expression of bcl2 and significantly increased the bax/bcl2 ratio. Increased bax induces apoptosis by stimulating the release of pro-apoptotic proteins such as cytochrome C from the mitochondrial intermembrane space to the cytoplasm [34]. As bcl2, an anti-apoptotic protein, can inhibit Ca^2+^ or bax-mediated cytochrome C release, a decrease in bcl2 plays an important role in inducing apoptosis [35]. These results suggest that the decreased expression of *ITGA7* could be associated with the increased expression of α-syn and apoptosis of dopaminergic cells. In addition, since the protein encoded by *ITGA7* is involved in the membrane and the aggregated form of α-syn promotes membrane destruction, an increase in α-syn expression due to a decrease in *ITGA7* expression may contribute to transmembrane pathogenesis. In particular, *ITGA7* may be involved in the mechanism by which α-syn acts on the membrane. Immunofluorescent analysis of the SN regions demonstrated that *ITGA7* and α-syn were localized near the membrane, and the MPTP group showed stronger α-syn expression and weaker *ITGA7* expression than the CTL group.

Considering that the increase in cell membrane α-syn expression leads to the destruction of dopaminergic cells [36], the increase in α-syn expression may be caused by a decrease in the expression of *ITGA7*, which is present in the cell membrane.

In the IHC results of this experiment, the expression pattern of *ITGA7* in the CTL group was quite similar to that of α-syn expressed in the MPTP group. The decrease in *ITGA7* expression induced by *ITGA7* siRNA in SH-SY5Y cells confirmed that the expression of α-syn was inversely increased. Based on these results, we believe that additional research on whether α-syn expression increases to replace the decrease in *ITGA7* expression is needed.

## 4. Materials and Methods

### 4.1. MPTP-Induced Parkinson’s Disease Mouse Model

The study protocol was approved by the Institutional Animal Care and Use Committee (IACUC) of Sangji University (IACUC 2018-6). Four-week-old male C57BL/6 mice (20–22 g; DBL, Daejeon, Korea) were used. The mice were divided into two groups: control (CTL) and MPTP groups. The mice in the CTL group were administered 0.9% (100 μL) saline intraperitoneally once every 24 h for 4 weeks. In the MPTP group, MPTP-HCl (20 mg/kg of free base) in 0.9% (100 μL) saline was intraperitoneally injected once a day for 4 weeks at 24-h intervals.

### 4.2. Cell lines and Cultures

Human-derived SH-SY5Y cells were grown at 37 °C in a humidified CO_2_ environment in 5% minimum essential medium (MEM; Welgen, Namcheon-myeon, Korea) supplemented with 10% fetal bovine serum (FBS; Lonza, Walkersville, MD, USA), 100 U/mL penicillin, and 100 mg/mL streptomycin.

### 4.3. *ITGA7* Small Interfering RNA

Stealth siRNA against *ITGA7* (5′-GAC AUG CAC UAC CUC GUC U-3′) and negative control duplexes (i.e., scrambled siRNA against *ITGA7*, 5-UUC UCC GAA CGU GUC ACG UTT-3′) were purchased from Bioneer Inc. (Daejeon, Korea). SH-SY5Y cells were treated with *ITGA7* siRNA for 24 h. Before siRNA transfection, SH-SY5Y cells were incubated in Opti-MEM medium (Gibco, Amarillo, TX, USA). Transfection reagent was used in a 3.5:1 transfection reagent-to-duplex RNA ratio (Promega, Madison, WI, USA) in Opti-MEM medium.

### 4.4. MPP+ Treatment

SH-SY5Y cells were treated for 18 h with 500 μM MPP + iodide (Sigma, St. Louis, MO, USA).

### 4.5. Western Blot

The SN and ST regions and SH-SY5Y cells were homogenized in radioimmunoprecipitation assay buffer (RIPA) on ice for 30 min for Western blot analysis. The lysate was centrifuged at 12,000 rpm at 4 °C for 20 min, and the protein concentration of the supernatant was measured using the bicinchoninic acid (BCA) method. The protein was separated by sodium dodecyl sulfate–polyacrylamide gel electrophoresis (SDS-PAGE) (4–15% Tris-Bis mini gel) and transferred to a polyvinylidene fluoride membrane. The membranes were blocked with 3% BSA at 37 °C for 1 h and 30 min and then incubated with anti-*ITGA7* (1:1000), anti-TH (1:2000), anti-bcl2 (1000), anti-bax (1: 1000), β-actin antibody (1:2000), or anti-α-syn (1:500) antibodies overnight at 4 °C. Subsequently, after washing three times for 15 min with TBST, the membrane was treated using horseradish peroxidase (HRP)-conjugated anti-mouse or anti-rabbit IgG antibody (1:5000) for 1 h. After washing with TBST, the membrane was visualized using a chemiluminescent substrate. Membrane band density was determined using ImageJ (https://rsbweb.nih.gov/ij/, accessed on 11 July 2021).

### 4.6. Immunohistochemistry

Mouse brains were fixed in 4% paraformaldehyde postfixed in 0.05 M sodium phosphate buffer containing 4% paraformaldehyde for 24 h at 4 °C, dehydrated with sucrose overnight at 4 °C, and then cryosectioned. Coronal sections of the brains (40 μm thickness) were cut using a cryomicrotome. Subsequently, each slice was treated with anti-integrin alpha 7 (1:1000, Santa Cruz Biotechnology, Santa Cruz, CA, USA), anti-TH antibody (1:2000, Santa Cruz Biotechnology), and anti-α-syn antibody (1:500, Santa Cruz Biotechnology), and then incubated overnight at 4 °C. The sections were then treated with biotinylated anti-mouse IgG, avidin-biotin-peroxidase complex, and diaminobenzidine hydrogen peroxide solution.

### 4.7. Immunofluorescence

After incubation with the primary antibodies and biotinylated anti-mouse IgG, each section was treated with fluorescein avidin DCS (Vector Laboratories, Burlington, ON, Canada). The sections were then treated with an avidin/biotin blocking kit and M.O.M mouse Ig blocking reagent (Vector Laboratories), after which they were stained with anti-α-syn or anti-*ITGA7* IgG at 4 °C overnight. Each section was treated with biotinylated anti-mouse IgG, followed by incubation with rhodamine D (Vector Laboratories, Burlington, ON, Canada). Photographic documentation was performed using a Nikon X-cite series 120Q microscope (Nikon, Tokyo, Japan), and the exposure parameters were the same for each group of samples.

### 4.8. Statistical Analysis

Statistical analysis was performed using Student’s *t*-test or analysis of variance (ANOVA) with SPSS 25 (SPSS Inc., Chicago, IL, USA) software. All values are shown as the mean ± standard error.

## 5. Conclusions

In this study, *ITGA7* expression was found to be reduced in the SN of an MPTP-induced Parkinsonism mouse model. In addition, the reduced expression of *ITGA7* may be related to the increased expression of α-syn and apoptosis of dopaminergic cells. Our results suggest that a decrease in *ITGA7* expression may be one of the causes of this increase in α-syn expression, which induces PD pathology.

## Data Availability

All data analyzed in this study are included in this published article.
